# Structural and Affinity Analyses of G-Quadruplex DNA Aptamers for Camptothecin Derivatives

**DOI:** 10.3390/ph6091082

**Published:** 2013-08-29

**Authors:** Hiroto Fujita, Yuri Imaizumi, Yuuya Kasahara, Shunsuke Kitadume, Hiroaki Ozaki, Masayasu Kuwahara, Naoki Sugimoto

**Affiliations:** 1 Graduate School of Science and Technology, Gunma University, 1-5-1 Tenjin-cho, Kiryu, Gunma 376-8515, Japan; 2 Division of Molecular Science, Faculty of Science and Technology, Gunma University, 1-5-1 Tenjin-cho, Kiryu, Gunma 376-8515, Japan; 3 Frontier Institute for Biomolecular Engineering Research (FIBER), Konan University, 7-1-20 Minatojima-minamimachi, Kobe 650-0047, Japan; 4 Faculty of Frontiers of Innovative Research in Science and Technology (FIRST), Konan University, 7-1-20 Minatojima-minamimachi, Kobe 650-0047, Japan

**Keywords:** DNA aptamer, G-quadruplex, camptothecin (CPT), circular dichroism (CD) spectroscopy, fluorescence titration, fluorescence polarization (FP) assay

## Abstract

We recently selected DNA aptamers that bind to camptothecin (CPT) and CPT derivatives from a 70-mer oligodeoxyribonucleotide (ODN) library using the Systematic Evolution of Ligands by EXponential enrichment (SELEX) method. The target-binding activity of the obtained 70-mer CPT-binding DNA aptamer, termed CA-70, which contains a 16-mer guanine (G)-core motif (G_3_TG_3_TG_3_T_2_G_3_) that forms a three-tiered G-quadruplex, was determined using fluorescence titration. In this study, truncated fragments of CA-70 that all have the G-core motif, CA-40, -20, -19, -18A, -18B, -17, and -16, were carefully analyzed. We found that CA-40 retained the target-binding activity, whereas CA-20, -19, and -18B exhibited little or no binding activities. Further, not only CA-18A but also the shorter length fragments CA-17 and -16 clearly retained the binding activity, indicating that tail strands of the G-quadruplex structure can significantly affect the target binding of G-quadruplex DNA aptamers. Further analyses using circular dichroism (CD) spectroscopy and fluorescence polarization (FP) assay were conducted to investigate the structure and affinity of G-quadruplex DNA aptamers.

## 1. Introduction

Although nucleic acid aptamers with high binding affinity and specificity for various molecular targets have been created using SELEX methods [1,2,3], many of them, DNA aptamers in particular, are known to contain G-quadruplex structures [4,5,6,7]. Therefore, it is generally considered that DNA aptamers with G-quadruplexes can target a broad range of molecular structures while retaining specificity. In other words, the structural diversity of G-quadruplexes, *i.e.*, parallel; anti-parallel; and mixed parallel/anti-parallel strand configurations with loops and tails of various sequence combinations, can achieve specific recognition of many molecules, even those with a similar scaffold [8]. However, the roles of loops and tails in target–aptamer complex formation have not clearly been elucidated and remain a subject of investigation [9,10,11,12].

As mentioned above, we recently recovered CA-70 and observed an unexpected phenomenon in its affinity analyses using fluorescence titration [13]. Namely, CA-20, -19, and -18B exhibited little or no binding activities, whereas CA-18A, CA-17, and -16 clearly retained the binding activity. The experimental data urged us to conduct further experiments to investigate how tail strands of G-quadruplexes influence the target binding of aptamers. In addition, studying the effects of tail strands on the binding activity of G-quadruplex DNA aptamers for small molecules may be of great importance for researches of G-quadruplex formation in genomic DNA, because the clinical potential of G-quadruplex binding with small molecules in down regulating gene transcription has recently been demonstrated [14,15]. Thus, here we determined which nucleotide residues in tail strands are critical for CPT-binding of DNA aptamers and verified structural differences between active and inactive fragments.

## 2. Experimental Section

### 2.1. General

**Figure 1:**
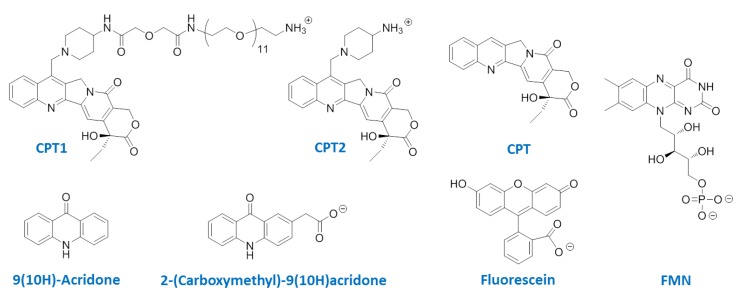
Chemical structures of analytes used in this study.

Ultraviolet-visible (UV-vis) spectra were measured using a U-3000 spectrophotometer (Hitachi High Technologies Corporation, Tokyo, Japan). Fluorescence spectra and fluorescence polarization were measured with a LS-55 spectrofluorophotometer (Perkin Elmer Japan Co., Ltd., Kanagawa, Japan). CD spectra were acquired with a JASCO J-820 spectrometer equipped with thermoelectrically controlled cell holders (JASCO Corporation, Tokyo, Japan). ODNs used were purchased from GeneDesign Inc. (Osaka, Japan) (Table 1). (*S*)-(+)-Camptothecin (CPT), 9(10*H*)-acridone, fluorescein, and polyoxyethylene sorbitan monolaurate (TWEEN^®^ 20) were purchased from Tokyo Chemical Industry Co., Ltd. (Tokyo, Japan). Riboflavin-5′-phosphate sodium (FMN) was purchased from Yamasa Corporation (Chiba, Japan). The seven-substituted camptothecin derivatives (CPT1 and CPT2) were synthesized from CPT according to the method reported in the literature [13] (Figure 1). 2-(Carboxymethyl)-9(10*H*)acridone was synthesized according to the method reported in the literature [16].

**Table 1:** Sequences and binding affinities of CA-70 and its fragments.

Fragment	Sequence *^a^*								*K*_d_ (µM) *^b^*		
									Analyte		
	Position	10	20	30	40	50	60	70	CPT1	CPT2	CPT
		|	|	|	|	|	|	|			
CA-70	GGTCAGCACGCTCCGGACTT **GGG**T**GGG**T**GGG**TT**GGG**GTACGGTGCGTAATGTGTCGCTGAGCCTGCCAAC								1.8 ± 0.3	3.6 ± 0.2	4.6 ± 0.3
CA-40	ACGCTCCGGACTT **GGG**T**GGG**T**GGG**TT**GGG**GTACGGTGCGT								3.0 ± 0.4	3.0 ± 0.2	5.6 ± 0.5
CA-23A	ACTT **GGG**T**GGG**T**GGG**TT**GGG**GTA								2.1 ± 0.9	n.d.	n.d.
CA-23B	TTTT **GGG**T**GGG**T**GGG**TT**GGG**GTA								>10	n.d.	n.d.
CA-20	T **GGG**T**GGG**T**GGG**TT**GGG**GTA								>10	>10	>10
CA-19	**GGG**T**GGG**T**GGG**TT**GGG**GTA								>10	>10	>10
CA-18A	T **GGG**T**GGG**T**GGG**TT**GGG**G								6.0 ± 0.8	3.4 ± 0.3	>10
CA-18B	**GGG**T**GGG**T**GGG**TT**GGG**GT								>10	>10	>10
CA-17	**GGG**T**GGG**T**GGG**TT**GGG**G								8.1 ± 0.9	8.9 ± 1.0	>10
CA-16	**GGG**T**GGG**T**GGG**TT**GGG**								1.8 ± 0.3	2.7 ± 0.3	4.2 ± 0.4

*^a^* Sequences are aligned in the 5′ to 3′ direction. Regions of G-quadruplex motif are marked with yellow boxes. *^b^ K_d_* values were determined by fluorescence titration with the assumption that fragments bind targets in a 1:1 ratio. n.d., not determined.

Buffer solutions were prepared using ultrapure water obtained using the Ultrapure Water system CPW-100 (Advantec Co. Ltd., Tokyo, Japan); 10 mM PBS buffer (pH 7.4) with 138 mM NaCl, 2.7 mM KCl, 2.5 mM MgCl_2_, and 0.05% TWEEN^®^ 20, and 20 mM Tris-HCl buffer (pH 7.2) with different KCl concentrations (0, 5, 10, 20, and 50 mM).

### 2.2. Job Plot Analysis

**Figure 2:**
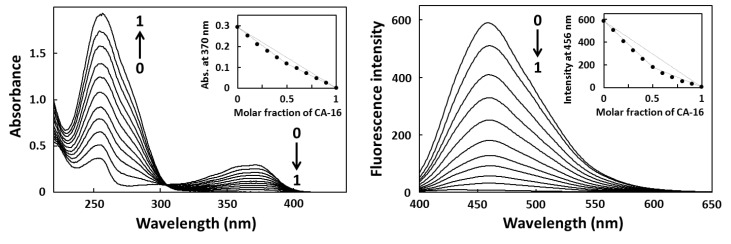
Continuous variation binding analysis (Job plot) of CPT1 using UV-vis spectroscopy (left) and fluorescence spectroscopy (right). Total concentration of CPT1 and CA-16 was maintained at 10 μM.

To determine the binding stoichiometry between the aptamer and target, a continuous variation binding analysis (Job plot) was performed [17,18]. First, CA-16 was dissolved in 10 mM PBS buffer to make a final concentration of 10 µM, and refolded by denaturing at 94 °C for 0.5 min, followed by cooling to 25 °C at a rate of 0.5 °C/min, while CPT1 was dissolved in 10 mM PBS buffer to make a final concentration of 10 µM. These solutions were mixed to maintain the total molar concentration (CA-16 and CPT1) at 10 µM, whereas the CA-16 mole fraction was varied at regular intervals from 0 to 1.0. After incubation at 25 °C for 30 min, the spectra of 11 sample solutions were measured using UV-vis spectroscopy and fluorescence spectroscopy to generate the Job plots (Figure 2).

### 2.3. Fluorescence Titration

**Figure 3:**
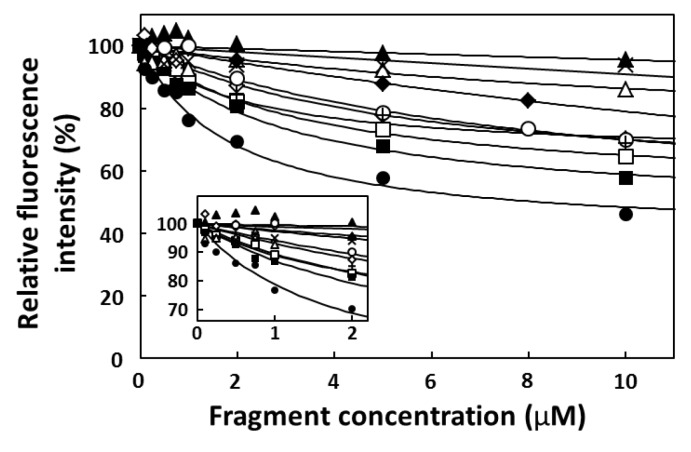
Titration curves for relative fluorescence intensity at 456 nm versus fragment concentration: CA-70 (open squares), CA-40 (closed squares), CA-23A (crosses), CA-23B (x-marks), CA-20 (open triangles), CA-19 (closed triangles), CA-18A (open diamonds), CA-18B (closed diamonds), CA-17 (open circles), and CA-16 (closed circles). CPT1 fluorescence without fragment was set at 100%.

Fluorescence titration of target (CPT1, CPT2, and CPT; final conc. 1.0 µM) with increasing concentrations of CA-70 and its fragments (final conc. 0–10 µM) was performed at 25 °C using LS-55. CA-70, -40, -23A, -23B, -20, -19, -18A, -18B, -17, and -16 were dissolved in 10 mM PBS buffer at appropriate concentrations (0–20 µM), and refolded by denaturing at 94 °C for 0.5 min and cooling to 25 °C at a rate of 0.5 °C/min. These solutions (50 µL each) were mixed with 50 µL solutions of target (CPT1, CPT2, and CPT; 2 µM) in 10 mM PBS buffer, respectively. Emission spectra of CPTs were obtained by exciting at 370 nm and monitoring fluorescence between 400 and 530 nm. In Figure 3, Y-axis indicates the relative fluorescence intensity (%) at 456 nm for CPT1 as an emission peak, where the intensity in the absence of fragments was set at 100%, and X-axis indicates fragment concentration. The relative fluorescence intensity (%) at 443 nm for CPT and at 456 nm for CPT2 was used to obtain the titration curves, respectively. The *K*_d_ values determined from the titration curves were listed on Table 1. To assess the binding specificity of CA-70, fluorescence titrations were also performed using the protocol described above and the following fluorophores: FMN, 9(10H)-acridone, 2-(carboxymethyl)-9(10*H*)acridone, and fluorescein (Figure 1).

### 2.4. FP Assay

**Figure 4:**
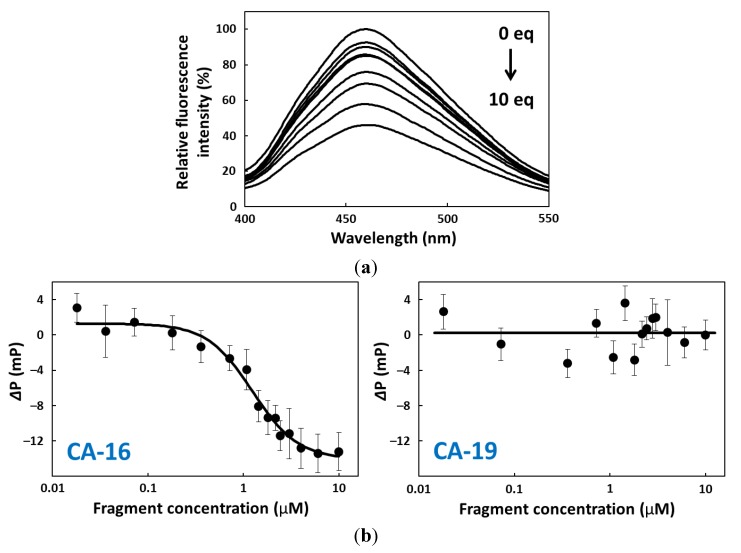
(**a**) Fluorescence spectra for CPT1 with varying CA-16 concentrations and excitation at 372 nm. CPT1 fluorescence without fragment was set at 100%. (**b**) Titration curve for CPT1 polarization versus fragment concentration: CA-16 (left) and CA-19 (right). Polarization was monitored at 456 nm using an excitation wavelength of 372 nm. *Δ*P = P − P_0_, where P is polarization with fragment, and P_0_ is polarization without fragment.

Fluorescence polarization measurements were also made for CA-16 and -19 affinity analyses [19,20]. CA-16 and -19 were dissolved in 10 mM PBS buffer at appropriate concentrations (0–20 µM), and refolded by denaturing at 94 °C for 0.5 min and cooling to 25 °C at a rate of 0.5 °C/min. These solutions (50 µL each) were mixed with 50 µL solutions of CPT1 (2 µM) in 10 mM PBS buffer, respectively. Using LS-55, fluorescence polarizations for each of the mixtures described above were recorded every 20–30 seconds for 10 min with excitation at 372 nm and monitoring at 456 nm at 25 °C. These measurements were performed three times in independent experiments. Thus, fluorescence polarization results for CPT1 (final conc. 1.0 µM) at different fragment concentrations (final conc. 0–10 µM) were generated as shown in Figure 4b.

### 2.5. CD Spectroscopy

**Figure 5:**
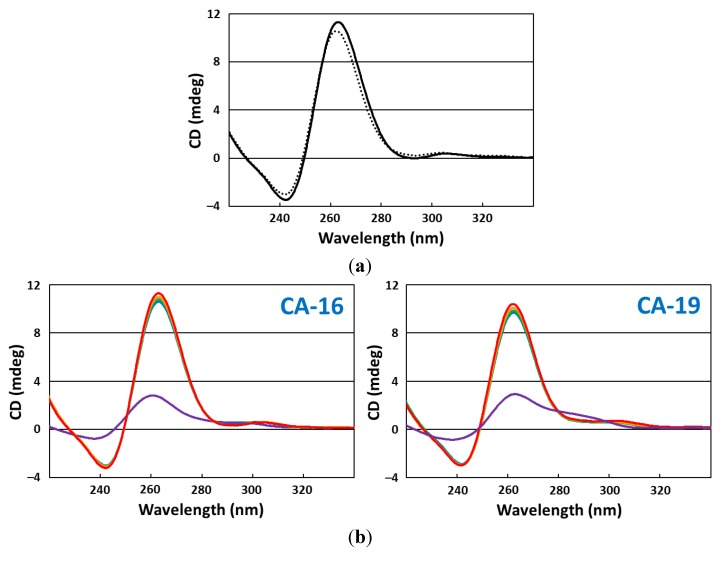
CD spectra for (**a**) CA-16 (solid line) and CA-19 (dashed line) in 10 mM PBS buffer, pH 7.4, and for (**b**) CA-16 (left) and CA-19 (right) in 20 mM Tris-HCl buffer, pH 7.2, and with different K^+^ concentrations: 0 mM (purple), 5 mM (blue), 10 mM (green), 20 mM (orange), and 50 mM (red).

CD spectra were measured over a wavelength range of 220–340 nm using a quartz cuvette with a 1.0-mm optical path length. The scanning speed was set at 100 nm/min, and the response time was 1 s. Each spectrum was an average of five measurements made at 25 °C. Before CD measurements, each fragment (CA-16 and -19) was dissolved in 20 mM Tris-HCl buffer (pH 7.2) for a 20 µM solution followed by refolding by denaturing at 94 °C for 0.5 min and cooling to 25 °C at a rate of 0.5 °C/min using the thermal cycler. Sample solutions containing 10 µM (final conc.) of fragment in 20 mM Tris-HCl buffer (pH 7.2) with different KCl concentrations (0, 5, 10, 20, and 50 mM) were prepared and analyzed (Figure 5). Similarly, the CD spectra of these fragments in 10 mM PBS buffer were measured.

### 2.6. CD Melting Curves

Ellipticity versus temperature profiles (melting curves) were measured at 265 nm in 0.1-cm path length cells. CA-16 was dissolved in 10 mM PBS buffer to make a final concentration of 10 µM, and refolded as described above. Sample solutions of CA-16 with or without CPT1 (10 µM) were incubated at 25 °C for 30 min before CD measurements. Melting curve experiments were performed from 25 °C to 95 °C at a heating rate of 0.25 °C/min. The melting temperatures (T_m_), *i.e.*, the midpoint temperatures of the unfolding process, were determined on the basis of sigmoid curves produced by fitting the data points [21,22,23].

## 3. Results and Discussion

**Figure 6:**
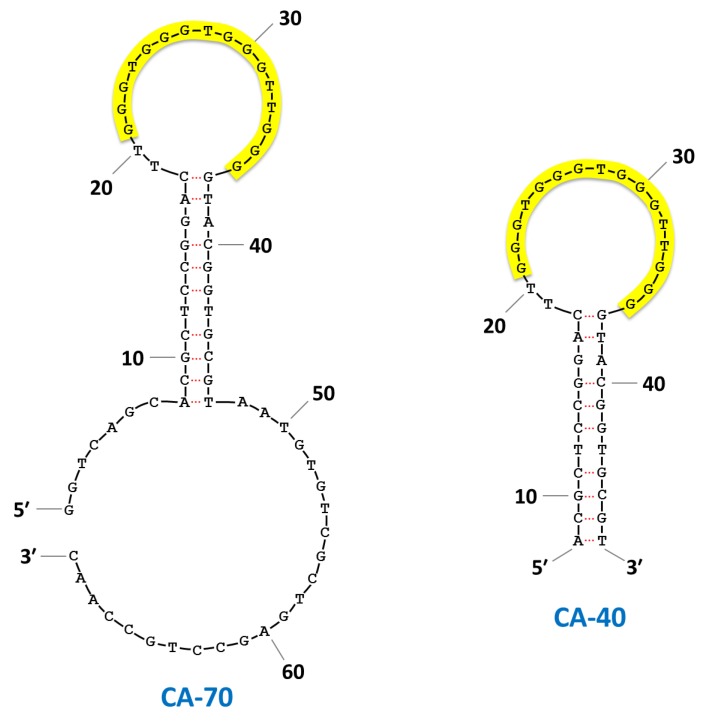
Predicted secondary structures of CA-70 and -40 using mfold-DNA folding form. The G-quadruplex motif was highlighted with yellow.

In our previous study, CA-70 was truncated into the 40-mer fragment CA-40 on the basis of secondary structure predictions by mfold performed at 37 °C in the presence of 100 mM Na^+^ [24,25] (Figure 6). As seen in the predicted structures, the 16-mer G-core sequence is located in a loop flanked by sequences that form a stem structure. Thus, we then further truncated CA-40 into CA-20 and -16, both of which contained the G-core sequence. The *K*_d_ values of CA-40 and -16 for CPT1 were 3.0 and 1.8 µM, respectively, which were comparable to that of non-truncated CA-70 (*K*_d_ = 1.8 µM), whereas CA-20 had little or no binding affinity for the target. First, we generated four new truncated fragments (CA-19, -18A, -18B, and -17) having the G-core sequence with a 3′-tail of 1–3 nucleotides and with or without a 5′-tail of a single nucleotide, and then compared their binding activities with each other.

The Job plot analysis determined the equimolar stoichiometry of the shortest CA-16 fragment that bound to the target CPT1 (Figure 2). Therefore, all *K_d_* values were determined on the basis of fluorescence titration with the assumption that fragments bind to the targets in a 1:1 ratio (Table 1 and Figure 3). The affinity analyses indicated that CA-17 retained the target-binding activity, but it was inferior to that of CA-16 because of the presence of a residue at position 37 (G^37^). Furthermore, compared with CA-17, both CA-18B and -19 showed much weaker binding activities because of the presence of one (T^38^) and two residues (T^38^A^39^) at the 3′-end, respectively. In contrast, CA-18A bound to CPT1 with a *K*_d_ value of 6.0 µM, which was comparable to that of CA-17. Thus, these results indicate that the addition of a residue to CA-16 at the 3′-end but not at the 5′-end sensitively affects the target-binding activity. It is possible that the binding site of CPT1 is near the 3′-terminus guanine base (G^36^) that is incorporated into the G-quartet planar structure [26]. Therefore, in case of CA-17 and -18A that retained certain binding activities despite the presence of G^37^, G^37^ can participate in G-quartet formation instead of G^36^ if G^34^, which is three nucleotide residues upstream of G^37^, is shifted into a loop of the G-quadruplex.

**Figure 7:**
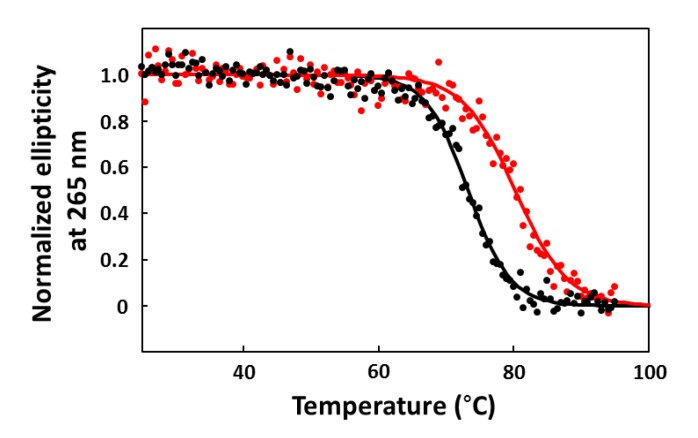
Melting curves for CA-16 in the presence (red) and absence (black) of CPT1, which were obtained by plotting the ellipticity at 265 nm against the temperature.

However, if this is the case, it remains unknown why CA-70 and -40 that have long 3′-tails retain the binding activity. Before addressing this issue, we had to ascertain the differences between active and inactive fragments in the binding activity, because the abovementioned observations were made using fluorescence quenching titration; the degree of quenching is not necessary consistent with the target-binding ratio. Therefore, we analyzed CA-16 and -19 by the FP assay and confirmed that the former has evident CPT1-binding activity but the latter does not (Figure 4). Then, we speculated that the extra 3′-terminus G^37^T^38^A^39^ may interfere with G-quadruplex formation; however, CD spectra showed that both CA-16 and -19 formed a parallel G-quadruplex in either buffer solution [27,28] (Figure 5). Also, the K^+^ dependency on G-quadruplex formation was confirmed (Figure 4b). A similar G-core sequence (AG_3_TG_4_AG_3_TG_4_) in a promoter region of c-Myc gene is known to form a three-tiered parallel G-quadruplex [29,30]. Therefore, it is conceivable that CAs can form parallel G-quadruplexes, although CAs have short loops consisting of 1–2 nucleotide residues. These parallel G-quadruplex cores are extremely stable. The T_m_ value of CA-16 was observed to be 73 °C in 10 mM PBS buffer (Figure 7). The T_m_ value was elevated by 80 °C when CPT1 associated with CA-16, indicating a significant contribution to the thermodynamic stability owing to π–π stacking interaction between the G-quartet plane and heterocyclic rings of the target.

**Figure 8:**
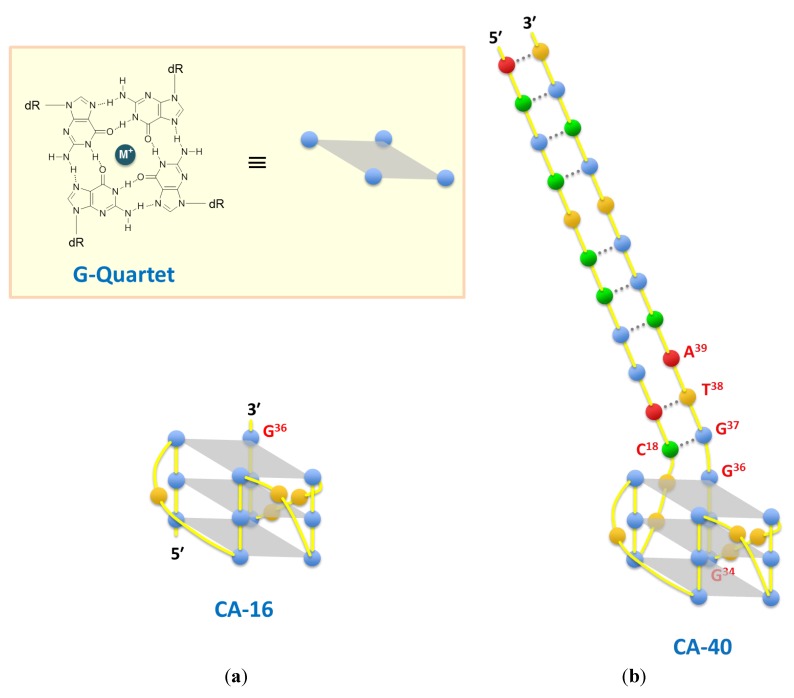
(**a**) Hydrogen-bonding pattern in a G-quartet (upper), and cartoon of a 16-mer parallel G-quadruplex, CA-16 (lower). (**b**) Cartoon of a putative conformation of CA-40. Base colors: red = adenine, blue = guanine, green = cytosine, and orange = thymine.

Overall, our experimental data indicated that the 3′-tail would not interfere with G-quadruplex formation but would interfere with target binding to the G-quartet plane involving G^36^. The first base of the 3′-tail may compete with CPT1 against space for binding on the adjacent G-quartet plane. However, in case of CA-70 and -40, the first base of the 3′-tail G^37^ pairs with C^18^ and is involved in forming the stem duplex. This may provide conformational stress on the whole structure, resulting in a bend in the joint of the stem and G-quadruplex and consequently create room for target binding on the G^36^-containing G-quartet plane (Figure 8b). To verify this hypothesis, we further generated two truncated fragments (CA-23A and -23B), having the G-core sequence with a 3′-tail of GTA and 5′-tail of ACTT or TTTT, and subsequently compared their binding activities. CA-23A can form two successive base pairs between both tails, whereas CA-23B cannot. As expected, CA-23A retained the binding activity, whereas CA-23B exhibited little or no binding activity (Table 1 and Figure 3). Thus, these data demonstrated the importance of C^18^–G^37^ base pairing in the aptamer-target interaction.

The binding specificities of active fragments were analyzed using CPT1, CPT2, and CPT (Figure 1). Except for CA-18A, fragments preferred targets in the following order: CPT1 > CPT2 > CPT. This was consistent with the binding specificity of the mother aptamer, CA-70 (Table 1). These results were reasonable because CPT1 was used for SELEX to find CPT-binding DNA aptamers. Although, the specificity of binding of CA-70 was not as specific as that of CPT-binding modified DNA aptamers reported previously [13], CA-70 could clearly distinguish CPTs from other aromatic compounds, such as 9(10H)-acridone, 2-(carboxymethyl)-9(10H)acridone, and fluorescein (*K*_d_ values of >10 µM), while it exhibited lower affinity for FMN (*K*_d_ = 8.5 µM). Thus, subtle distortions on the G^36^-containing G-quartet plane in addition to electrostatic interactions and steric hindrance with base-, sugar-, and phosphate moieties adjacent to G^36^ could form the binding site and enable specific binding of small molecules.

## 4. Conclusions

Unlike antibody, the G-quadruplex does not appear to form complex tiny cavities to fit small ligands with large structural variations. However, many compounds that can bind to the G-quadruplex have been reported and sequences with the G-quadruplex motif can discriminate between small molecules based on specificity to a certain extent. We believe that the insights derived from our results will provide clues for devising a more universal approach for small ligand recognition using G-quadruplex DNA. Small molecules that target the G-quadruplex of a particular gene can be rationally designed and used in future for artificial gene regulation for bioresearch, bioengineering, and chemotherapy. 
